# Understanding the Adsorption of Peptides and Proteins onto PEGylated Gold Nanoparticles

**DOI:** 10.3390/molecules26195788

**Published:** 2021-09-24

**Authors:** Yasiru Randika Perera, Joanna Xiuzhu Xu, Dhanush L. Amarasekara, Alex C. Hughes, Ibraheem Abbood, Nicholas C. Fitzkee

**Affiliations:** 1 Department of Chemistry, Mississippi State University, Starkville, MS 39762, USA; yrp7@msstate.edu (Y.R.P.); xx79@msstate.edu (J.X.X.); dla216@msstate.edu (D.L.A.); ach436@msstate.edu (A.C.H.); 2 Department of Chemistry, University of Arkansas at Little Rock, Little Rock, AR 72204, USA; ibraheemabbood12@gmail.com

**Keywords:** NMR spectroscopy, gold nanoparticles, PEGylation, adsorption, passivation

## Abstract

Polyethylene glycol (PEG) surface conjugations are widely employed to render passivating properties to nanoparticles in biological applications. The benefits of surface passivation by PEG are reduced protein adsorption, diminished non-specific interactions, and improvement in pharmacokinetics. However, the limitations of PEG passivation remain an active area of research, and recent examples from the literature demonstrate how PEG passivation can fail. Here, we study the adsorption amount of biomolecules to PEGylated gold nanoparticles (AuNPs), focusing on how different protein properties influence binding. The AuNPs are PEGylated with three different sizes of conjugated PEG chains, and we examine interactions with proteins of different sizes, charges, and surface cysteine content. The experiments are carried out in vitro at physiologically relevant timescales to obtain the adsorption amounts and rates of each biomolecule on AuNP-PEGs of varying compositions. Our findings are relevant in understanding how protein size and the surface cysteine content affect binding, and our work reveals that cysteine residues can dramatically increase adsorption rates on PEGylated AuNPs. Moreover, shorter chain PEG molecules passivate the AuNP surface more effectively against all protein types.

## 1. Introduction

When nanoparticles (NP) encounter a biological environment, biomolecules will spontaneously adsorb to the NP surface, forming a biomolecular corona. For many biomedical applications, the NP surfaces are designed to elicit the adsorption of biomolecules for bioimaging and biosensing [1]. On the other hand, some NP surfaces are fabricated to limit the adsorption of biomolecules, preventing recognition by the human body [2]. This is especially true for gold nanoparticles (AuNPs) that must pass through the blood–brain barrier or otherwise avoid clearance from the immune system [3,4]. However, when administered intravenously [5], AuNPs are rapidly removed from the circulation and accumulate mainly in the liver and the spleen due to the opsonization and recognition by phagocytes [3,6]. Many studies attempt to reduce the undesirable uptake of AuNPs by modifying the physicochemical properties of AuNP such as size, surface charge, hydrophilicity, and surface functionality [4,7,8,9,10,11].

A well-known method to avoid phagocytosis is to functionalize NP surfaces with polyethylene glycol (PEG), also called PEGylation [12,13]. There are numerous studies indicating that PEG-coated AuNPs reduce the protein corona formation and increase the retention time of the NPs in the circulatory system [14,15,16]. The passivating effect of PEG on AuNPs is thought to be conferred due to a reduced tendency for protein binding to a PEGylated surface [17,18]. As this biocorona layer of bound proteins is reduced, the immune system does not recognize the AuNPs, and therefore PEGylated NPs will persist [19,20]. In addition, PEG-passivated NPs are widely used as drug carriers as the solution exposed termini can be modified with a drug [21,22,23,24]. As an example, doxorubicin can be effectively attached to PEG-coated AuNPs to target cancer cells for treatment [25].

In practice, there are numerous approaches to PEGylation, and size, terminal modification, and charge can all be modified [26,27,28]. PEGylation is conducted using several unique techniques including detritylating PEG derivatives, covalent attachment, entrapment, or AuNP surface adsorption of PEG chains. The effect of the PEGylation depends on the PEG molecular weight, polymer chain architecture, and PEG surface density on the NP surface [29,30]. For example, a high density of PEG favors an extended “brush” conformation, which is more effective at passivation than the “mushroom” conformation adopted during less dense PEGylation [16]. This improved passivation likely results from a shift in PEG dynamics, with more constrained motions favoring improved passivation [31]. These findings are of great relevance for studying protein adsorption onto PEGylated spherical nanoparticle surfaces, and the protein adsorption resistance on the NP surfaces can vary drastically depending on the PEGylation strategy.

In addition to the properties of PEG itself, protein properties are also likely very important in determining the effectiveness of passivation, and some proteins are better than others at circumventing passivation strategies. For example, while PEGylation reduces nonspecific binding overall, gel electrophoresis and mass spectrometry identify differences in how individual proteins interact with poly(DL-lactide-co-glycolide) (PLGA) nanoparticles upon passivation with PEG [32]. Another recent study by Blume et al. used the protein corona of PEGylated superparamagnetic iron oxide NPs as a probe for proteomics analysis, demonstrating that PEG does not block binding from all proteins equally [33]. Both of these studies were carried out at shorter incubation periods, under 12 h, in which PEG exhibits excellent passivating characteristics; nevertheless, some proteins are clearly able to bind PEGylated NPs. At longer timescales, the problem of protein binding becomes more pronounced, especially at low PEG densities [34,35]. Some nanoparticle formulations persist for > 24 h in the body [36], and therefore characterizing how blood proteins interact with PEG-coated NPs is an important aspect of drug delivery optimization. In particular, understanding how different biomolecular properties influence PEGylated NP binding could lead to better passivation strategies, or it could reveal insight into why some NP systems resist passivation.

In this study, we have quantitatively characterized several different peptides and proteins as they adsorb to PEGylated AuNPs. Both small peptides and larger proteins are studied, and the presence of cysteine residues is also systematically investigated. The AuNPs are coated with 5K, 10K, and 30K thiolated PEG, allowing us to probe different levels of PEG coverage. Using NMR spectroscopy [37,38,39], we have quantified the kinetics of and final stoichiometry of biomolecular adsorption to PEGylated AuNPs, and we discuss trends and physical properties that lead to efficient binding to PEGylated AuNP surfaces.

## 2. Results

### 2.1. PEGylation of Gold Nanoparticles and Protein Selection

**Scheme 1:**
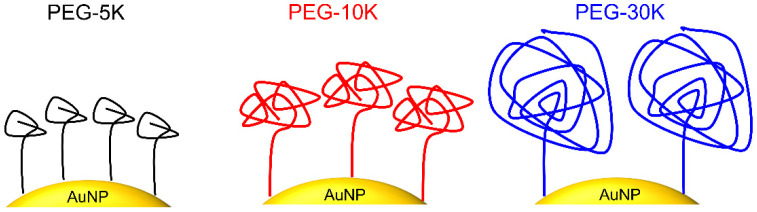
Conformational model of PEG-SH binding to an AuNP surface.

Prior to protein adsorption, AuNPs were incubated with an excess amount of different thiol PEG compounds (PEG-SH) to fully passivate the surface. The attachment of PEG-SH on AuNP was characterized using UV-Vis spectroscopy, Zeta potential and dynamic light scattering (DLS) (Table 1). An increasing hydrodynamic diameter was observed when larger PEG-SH molecules are bound to the AuNP surface. Zeta potentials of all AuNP-PEG conjugates increase from −38 ± 3 mV to around −20 mV, indicating the negatively charged citrate-capped AuNP surfaces are all saturated with PEGs. Transmission electron microscopy (TEM) is consistent with 15-nm AuNPs, showing no AuNP aggregation during synthesis of AuNP-PEGs. Visualization of the PEG in TEM is difficult due to the formvar grids used in TEM experiments, and the PEG layer itself was not consistently observed (Figure S2, Supporting Information). The localized surface plasmon resonance (LSPR) peak of AuNPs at 520 nm has a decreasing degree of redshift of 3, 2, and 1 nm for 5K-PEG-SH-, 10K-PEG-SH-, and 30K-PEG-SH-coated AuNP, respectively (Table 1 and Figure S1, Supporting Information). This decrease in LSPR redshift as PEG size increases was previously reported [40], and the larger redshift for shorter PEGs likely arises from a mushroom-like structure of PEG: the end with a terminal thiol group binds to AuNP through a strong gold-thiolate bond, and the long PEG polymer chain adopts a random coil shape [14,40]. Based on this model, it is hypothesized that shorter PEG-SHs are grafted more densely on the AuNP than longer ones, which explains why PEG-SH-5K can most effectively change the dielectric constant of the immediate medium surrounding AuNP surface, and therefore induces the largest LSPR redshift. Indeed, the experimentally measured PEG density on these AuNP-PEG conjugates decreases from 0.96 ± 0.01 to 0.57 ± 0.01 PEG/nm^2^ as PEG size increases from 5K to 30K (Table 1), supporting the predicted PEG conformational model on the AuNP surface. This is consistent with prior studies examining the trend of PEG-SH coverage on AuNPs [34,41]. Scheme 1 depicts the PEG-SH conformations on AuNP surface and PEG density decreases with larger PEG-SHs.

**Figure 1:**
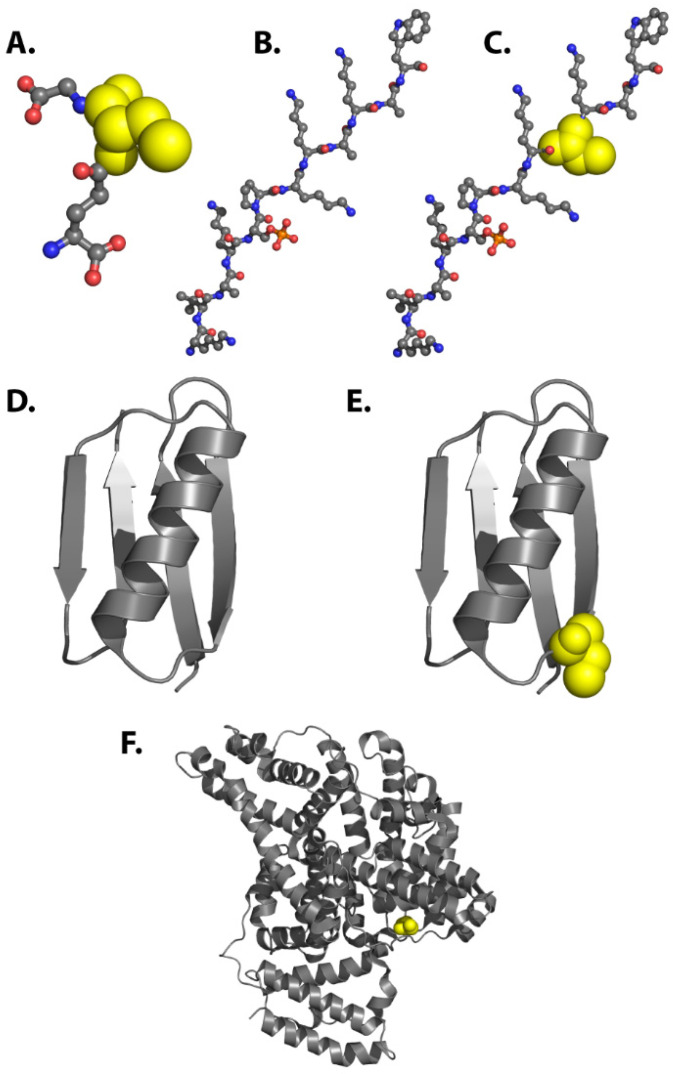
Peptides and proteins used in this study. (**A**) Glutathione peptide (GSH), (**B**) H1.5 peptide, (**C**) H1.5-Cys peptide, (**D**) Wild-type GB3 protein (WT GB3, PDB 2OED), (**E**) K19C GB3 variant (K19C GB3), and (**F**) bovine serum albumin (BSA, PDB 3V03 chain A). Peptides employ a ball-and-stick representation, whereas proteins are represented with cartoons. Cysteine residues that do not form intramolecular disulfide bonds are shown as yellow spheres in space filling representation. Note that peptides and proteins are not drawn to scale.

Permeability of a biomolecule into the PEG chains is essential for its binding onto AuNP surface. In this work, protein size and surface thiol groups are hypothesized to be the key factors that determine permeability. This is for two reasons: First, smaller molecules are expected to diffuse into PEG chains more easily than larger ones. Second, thiol groups have a particularly strong affinity towards AuNP, which facilitates binding through formation of gold-thiolate bond [42,43]. Therefore, representative molecules are selected to be glutathione (GSH), the H1.5 peptide (with and without cysteine), wild-type GB3, the K19C GB3 variant, and bovine serum albumin (BSA) (Figure 1, Table 2). GSH is the smallest construct with only one thiol group, whereas the H1.5 peptide, which is phosphorylated and derived from a histone H1.5 segment, lacks a thiol. H1.5-Cys is identical to H1.5, but it contains a Cys residue at the 9th position. Larger protein candidates include WT GB3, which lacks cysteine residues, and K19C GB3, which contains one cysteine at position 19. BSA is the largest protein, and while it contains 35 Cys residues, only one of these is readily available to bond; the rest form disulfide bonds and are buried within the folded structure of the protein. The estimated net charge of each protein, assuming model compound pK_a_ values, is also presented. PEGylated AuNPs retain a negative ζ-potential, suggesting that more basic proteins may be favored [44]. Although the proteins selected do not exhaustively probe protein charge, the H1.5 peptide is highly basic and could potentially indicate whether electrostatic interactions influence how efficiently proteins can permeate a PEG layer.

### 2.2. Adsorption of Small Peptides onto PEGylated AuNPs

**Figure 2:**
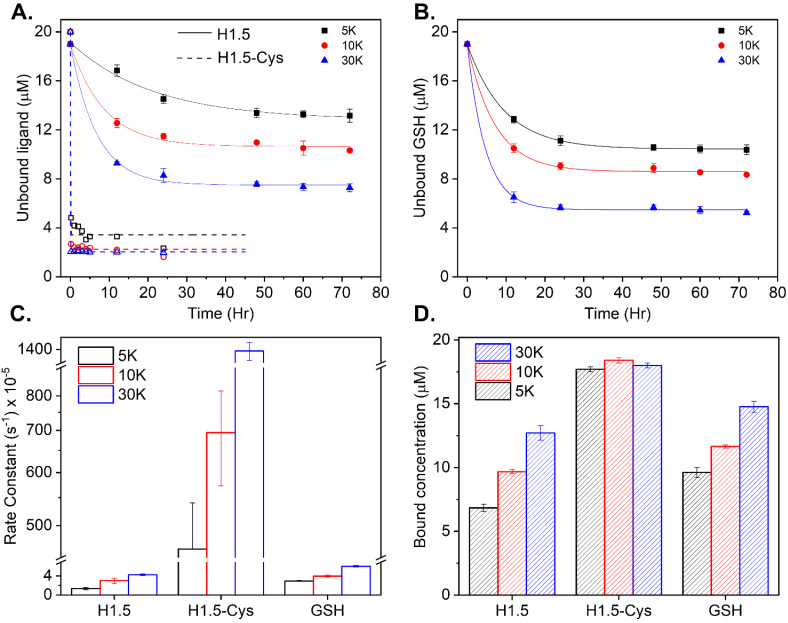
Adsorption of small peptides onto PEGylated AuNPs. (**A**) Free H1.5 (closed symbols, solid lines) and H1.5-Cys (open symbols, dashed lines) concentrations vs. time in the presence of 5K, 10K, and 30K PEGylated AuNPs during the 0–72 h incubation period. The kinetic study for H1.5-Cys was performed for only 24 hrs due to its fast adsorption kinetics. (**B**) GSH adsorption to PEGylated AuNPs during the same time duration. The curves in (**A**,**B**) are a pseudo-first-order kinetics fit, and the error bars are the standard error of the mean. (**C**) Adsorption rate constants for H1.5 and GSH for different PEGylated AuNPs obtained from the pseudo-first-order kinetic fit. (**D**) Bound concentration at the final time point of H1.5 and GSH for different PEGylated AuNPs after 72-hour incubation. The apparent adsorption rates and bound concentrations of all ligands used in this work are summarized in Table S1 (Supporting Information). Error bars in panels (**C**,**D**) represent uncertainties calculated from a weighted least squares minimization of the reduced chi-squared performed in OriginPro 2021b (Northampton, MA, USA).

We first monitored the adsorption of three small peptides onto PEG-SH coated AuNPs, and their adsorption rate constants and adsorbed amount are obtained for comparison (Figure 2A,B). The PEG size affects the adsorption rate constants as well as the final amount of peptides adsorbed onto AuNPs. All peptides are adsorbed fastest and in the greatest amount for AuNPs coated with 30K-PEG-SH, with adsorption rate and amount decreasing with smaller PEG size (Figure 2C,D). This indicates these molecules can penetrate more easily into the PEG layers formed with larger PEGs, and the AuNPs functionalized with larger PEGs have more available surface area for additional ligand binding. This observation is consistent with the grafting density of PEG-SH on AuNP, which is limited to an extent by the size of the coil-shaped PEG chains. The use of larger PEG-SH molecules results in less densely grafted PEG on AuNP, which facilitates diffusion of ligands into the PEG chains and leaves more unoccupied AuNP surface for binding [34]. Another observation is that GSH is adsorbed faster and in a larger amount than H1.5 on each type of AuNP conjugate. This is attributed to the smaller size of GSH (0.3 kDa) as compared to H1.5 (1.4 kDa), and high affinity afforded by a thiol group in GSH in contrast to no thiol group in H1.5.

Interestingly, incorporating a Cys residue into the H1.5 peptide dramatically enhances this peptide’s affinity toward PEGylated AuNPs (open symbols, Figure 2A). H1.5-Cys is identical to H1.5, except that it contains a cysteine residue in the 9th position (A9C). In all of our experiments, near-complete binding of H1.5-Cys occurred within fifteen min—the dead time of our measurements (Figure 2A). While apparent rate constants could be fit for H1.5-Cys, these are lower-bound estimates, and binding of H1.5-Cys could be faster. In addition, the amount of H1.5-Cys bound was higher than H1.5 for all sizes of PEGylated AuNPs (Table S1, Figure 2D). Apparently, the H1.5-Cys peptide is able to readily penetrate the PEG layer and form a stable thiolate bond. Nearly all of the H1.5-Cys peptide can be adsorbed, suggesting that PEG is not a particularly good passivating compound for small, basic peptides containing thiol groups. 

**Scheme 2:**
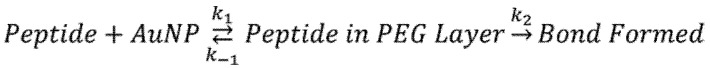
Kinetic scheme for peptide interaction with PEGylated AuNPs.

All of these experiments were performed under conditions with no added salt, so screening of electrostatic interactions should be minimal. The balance of size, electrostatics, and the presence of a thiol can be understood using the following simple scheme (Scheme 2):

In this scheme, the peptide is able to penetrate the PEG layer in a fast-equilibrium process ($K_{1}=k_{1}/k_{-1})$. This equilibrium is altered by electrostatics, which will favor penetration into the PEG layer for more basic peptides such as H1.5 (increasing $K_{1}$). It is feasible that the rate constant $k_{1}$ is sensitive to the peptide and to the density of the PEG layer. For example, smaller peptides (such as GSH) are able to penetrate the PEG layer more rapidly (increasing $k_{1}$), and denser PEG layers will likely decrease the speed of penetration (reducing $k_{1}$). In our work, $k_{2}$ is primarily dependent on whether a gold-thiolate bond can occur between the AuNP surface and a Cys residue. Other types of bonds are possible between proteins and Au surfaces, including amines and carboxylates [45,46,47,48], but the presence of a Cys residue should strongly increase the rate $k_{2}$. Here, our measured rate constants ($k_{obs}$) are approximately equal to $k_{obs}\approx K_{1}k_{2}$, a scenario that is analogous to EX2 conditions for hydrogen exchange ($k_{-1}\gg k_{2}$) [49]. Elucidating specific values for $K_{1}$ and $k_{2}$ is challenging, but our data qualitatively support this scheme. H1.5 is highly basic (larger $K_{1}$), but it is larger than GSH (slower $k_{1}$) and lacks a thiol (slower $k_{2}$), therefore it exhibits the slowest adsorption kinetics. H1.5-Cys contains a thiol (fast $k_{2}$), and therefore it binds very quickly, within the first 15 min of mixing. GSH, which is acidic (weaker $K_{1}$) and contains a thiol (fast $k_{2}$), is intermediate between H1.5-Cys and H1.5. In this scheme, the ratio of $k_{obs}$ for H1.5-Cys and H1.5 should correspond to the ratio of $k_{2}$ for a thiol attachment and $k_{2}$ for a non-thiol attachment (Equation (1)):

(1) $$\frac{k_{obs,\;H1.5-Cys}}{k_{obs,\;H1.5}}\approx \frac{k_{2,\;thiol}}{k_{2,non-thiol}}$$

Moreover, this ratio should be roughly constant, and indeed it is (350±80, 230±60, and 320±20 for 5K, 10K, and 30K, respectively). Our data therefore support a model where the peptides penetrate the PEG layer frequently, temporally sampling conformations near the AuNP-PEG interface, but adsorption only occurs occasionally when direct contact is made with the gold surface (enabling a more stable bond to occur).

### 2.3. Adsorption of Larger Proteins onto PEGylated AuNPs

**Figure 3:**
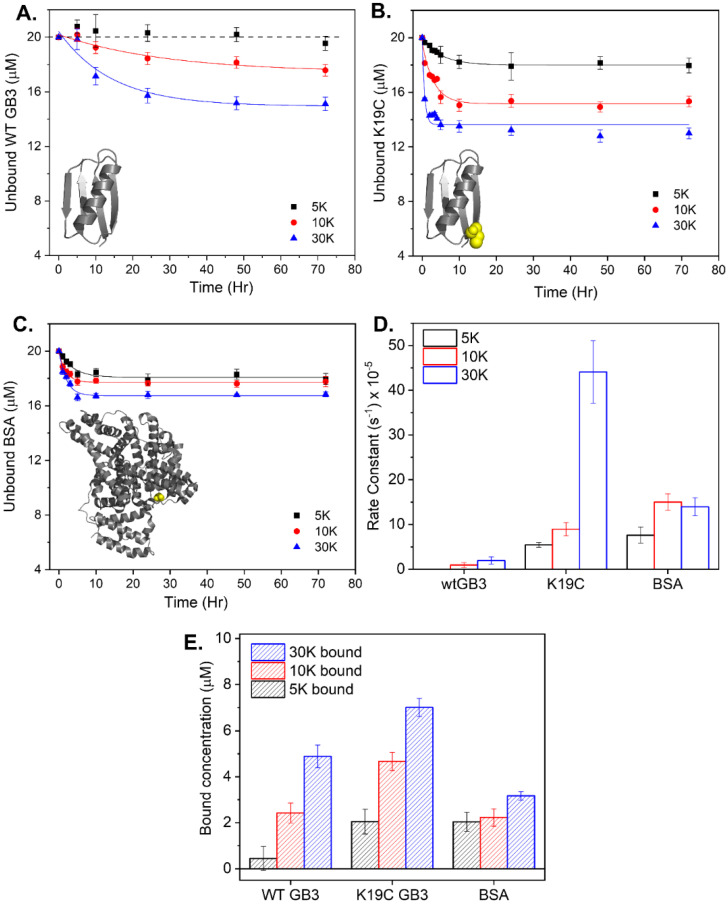
Adsorption of proteins onto PEGylated AuNPs. (**A**) Unbound WT GB3, (**B**) K19C GB3, and (**C**) BSA concentration to 5K, 10K, and 30K PEGylated AuNPs during the 0–72 h incubation period. The curves are the pseudo-first-order kinetics fit, and the error bars are the standard error of the mean. (**D**) Pseudo-first-order adsorption rate constants and (**E**) bound concentration for WT, K19C GB3, and BSA for different PEGylated AuNPs. Error bars in panels (**D**,**E**) represent uncertainties calculated from a weighted least squares minimization of the reduced chi-squared performed in OriginPro 2021b (Northampton, MA, USA).

To explore this scheme, we next tested the adsorption of proteins of varying sizes and thiol group content. The adsorption of the selected proteins is tight and in slow exchange timescale with no line broadening, rendering their NMR signals invisible upon attachment to the AuNP surface. Therefore, the remaining protein NMR signals correspond to the unbound protein amount [38,50]. Examples of quantifying protein unbound concentrations using 1D NMR for BSA and 2D NMR for K19C GB3 are presented in Figures S3 and S4, Supporting Information. As compared to the H1.5 peptide (1.4 kDa), WT GB3 has a size of 6.2 kDa. During the 72 h of our experiment, no WT GB3 was detected to adsorb at a statistically significant level onto 5K-PEG-AuNPs, and only a small amount of this protein penetrated into 10K- and 30K-PEG-AuNPs (Figure 3A,E). Specifically, 10% and 25% of the original 20 μM WT GB3 sample was adsorbed on the 10K and 30K PEG-SH molecules, respectively. For comparison, the H1.5 peptide reached ~60% adsorption after a similar incubation period on 30K-PEG-AuNPs. This suggests that, in the absence of high affinity thiol groups, small molecular size is critical for diffusion into the PEG layers, especially the denser PEG layer formed by smaller PEGs.

In contrast, when one cysteine residue is introduced into GB3 (K19C GB3), both adsorption amount and adsorption rate increase significantly for all types of PEGylated AuNPs (Figure 3). For example, while the adsorption of WT GB3 on 5K-PEG-AuNPs was barely within the limits of detection for our experiment over 72 h, 2.1 ± 0.5 µM K19C GB3 is able to penetrate and bind onto these nanoparticles after only 10 h incubation. This difference becomes smaller as the PEG size is increased. While greater adsorption is always observed for K19C GB3, 10K-PEG-AuNPs allow nearly twice as much binding of K19C vs. WT GB3, but 30K-PEG-AuNPs allow only 1.4 times as much binding. In addition to increasing the final amount of adsorbed protein, introducing a cysteine residue also increases the rate at which adsorption occurs. We could not determine a pseudo-first order rate constant for WT GB3 adsorption to 5K-PEG-AuNPs, but rate constants could be reliably fit for the larger PEG sizes. Indeed, the calculated adsorption rate constants of K19C GB3 for both 10K-PEG-AuNP and 30K-PEG-AuNP are ~9 and ~23 times larger, respectively, than those measured for WT GB3 (Figure 3D).

In the context of Scheme 2, GB3 is apparently able to penetrate the PEG layer, and it can penetrate 10K and 30K PEG layers more efficiently than 5K PEG. Previously, it was theorized that adsorption isotherms tend to become independent of PEG length beyond 2000 Da (50 monomer units) for lysozyme and fibrinogen [51,52]. Both lysozyme (14 kDa) and fibrinogen (300 kDa) are much larger than GB3 (6.2 kDa), and for GB3 we see a size dependent effect. In addition, taking the ratio $k_{obs}$ for K19C GB3 and $k_{obs}$ for WT GB3 does not appear to produce a constant value as it did with H1.5 (9±4 for 10K vs. 23±10 for 30K), suggesting that the complexity of larger proteins complicates Scheme 2. This may be as the size of GB3 is similar to the size of the PEG molecules themselves, and reorientation in the PEG layer becomes more difficult when the protein size is similar to the passivating PEG. As the PEG size increases, the attachment density decreases (Table 1), and GB3 is more easily able to slip through the passivating layer. Thus, the size and shape of the protein relative to the PEG density will likely influence whether the first step in scheme two is fast-to-equilibrium. Additional work is needed to explore this hypothesis; nevertheless, these results highlight the importance of free and surface cysteine (thiol) groups in protein adsorption onto PEGylated AuNP. The high affinity of thiol towards gold not only accelerates the adsorption rate of large biomolecules, but also increases the final bound amount.

On the other hand, BSA has a size ~10 times that of the GB3 protein. With the same number of free surface thiol groups as K19C GB3, the size effect of protein adsorption on PEGylated AuNP can be examined. Previous experiments using fluorescence spectroscopy found that BSA was buried inside the 10K PEG layer on an AuNP [14,26]. Our studies indicate that, due to the size of BSA, only a small concentration of BSA is adsorbed by all three types of PEGylated AuNP surfaces. The maximum adsorption is again observed for the 30K-PEG-AuNPs. The same trend that larger PEG promotes greater adsorption is observed, although the difference between 5K PEG and 30K PEG is markedly less for BSA vs. K19C GB3. Interestingly, even large proteins are able to penetrate relatively dense monolayers of PEG on an AuNP surface. While the amount of adsorbed protein is small, the value is reproducible, and such a small number may be able to alter the immune response to an engineered nanoparticle, especially if those nanoparticles experience long circulation times.

For comparison, the binding of these biomolecules onto non-PEGylated AuNPs is extremely fast and results in higher binding capacities. Previous studies show that ~90% of protein binding to citrate-coated AuNPs occurs in the initial ~5 min, and adsorption is completed within an hour [38,39]. The binding capacities (molecules per AuNp) for GSH, WT GB3 and BSA on bare AuNPs were determined to be 1430±90, 177±20, and 30±10, respectively [37]. In contrast, it takes 5–20 h for GSH, WT GB3, and BSA to reach their maximum binding capacities (molecules per AuNP) of 123±2, 41±4, and 123±2, 26±2, respectively on AuNP-PEG-30K. The binding rates and final amounts are even lower on AuNP-PEG-5K and AuNP-PEG-10K.

## 3. Discussion

The observation that proteins can penetrate PEG-passivated surfaces is not new [29,30]; however, systematic studies to identify which protein properties favor fouling of passivated AuNPs can be useful in improving strategies for limiting protein binding. Here, we design a simple test that examines how protein size, charge, and surface cysteine content influences adsorption for several different PEGylation densities on AuNPs, holding the size of the core AuNP constant. Our approach employs a newly developed external NMR referencing method for quantifying protein binding to AuNPs in situ, and our observations do not require displacement of proteins or treatment of the AuNPs [38]. This approach enables straightforward measurements of adsorption kinetics, provided no line broadening is observed in the biomolecular NMR spectra. We observe that both the adsorption rate and the number of biomolecules adsorbed onto PEGylated AuNPs increase as the size of PEG-SH increases, from 5K-, 10K-, to 30K-PEG-AuNPs. This validates previous observations that smaller PEGs have a better passivation effect on AuNPs due to their higher grafting density, making diffusion of biomolecules into the dense PEG polymer chains less efficient, and leaving less AuNP surface for biomolecule loading [16,34,51,52,53]. On the contrary, larger PEG can stabilize AuNPs as well as allow biomolecules to diffuse efficiently and bind to the AuNPs. With all peptides and proteins tested in this work, maximum binding for 30K-PEG-AuNP is attained within several hours. These results should provide insights into how to select PEG size for different applications of PEGylated AuNPs.

Comparing the adsorption behaviors of H1.5, GSH, and WT GB3 onto PEGylated AuNPs, we conclude that molecules with small sizes (significantly smaller than the PEG size) can efficiently penetrate the PEG layers, rapidly sampling conformations near the surface as shown in Scheme 2. The presence of thiol groups for small peptides can dramatically enhance adsorption. Once the protein becomes larger than the PEG itself, however, the situation is more complex and likely depends on the protein itself. Surface Cys residues are still important, as demonstrated by the difference between WT and K19C GB3. Steric hindrance from the densely populated PEG chains is effective at reducing protein binding, but the introduction of only one cysteine residue allows even weakly associated proteins to adsorb to the AuNP surface. Even BSA, which is approximately 10 times the size of WT GB3, can be bound to 5K-PEG-AuNP, the PEGylated AuNP with highest grafting density. The survey of these large biomolecules demonstrates high affinity groups are required for large molecules to be loaded onto PEGylated AuNPs.

In conclusion, our study indicates that larger PEG molecules are less effective in passivating biomolecules molecules with small sizes (GSH and H1.5) and macromolecules with thiol groups (H1.5-Cys, K19C GB3, and BSA). Larger PEG constructs adopt a “mushroom”-like structure, leaving voids in between the PEG chains [16]. Due to this mushroom-like structure, the surface density of larger PEG on AuNPs is lower than the short PEG chains. This phenomenon allows the proteins to easily penetrate the PEG layer. Shorter PEG chains are much more effective in passivating the AuNP surface from biomolecules, but the presence of surface cysteine residues appears to nullify the passivating effect, especially at timescales greater than 12 h, which are relevant for the pharmacokinetics of AuNP-based therapeutics.

## 4. Materials and Methods

### 4.1. Synthesis of Citrate-Stabilized Gold Nanoparticles

Spherical 15 nm AuNPs were synthesized via citric acid reduction using principles of the Turkevic synthesis method [54,55]. Tetrachloroauric Acid (HAuCl_4_) and sodium citrate dihydrate were purchased from Sigma-Aldrich. After 100 mL of 0.3 mM HAuCl_4_ had been heated to boiling, 2 mL of 34 mM sodium citrate solution was immediately mixed with the gold solution. This mixture was stirred with heating for an additional 20 min before being cooled to room temperature. The cooled solution was then centrifuged for 45 min at 9000× *g*. The concentrated AuNP sample was then extracted and sonicated for 6 min (in 1-min intervals) at a power level of 1 on a Branson sonicator. The sonicated sample was characterized via UV-visible spectroscopy, dynamic light scattering, and transmission electron microscopy for size and conformity [37]. For 15 nm AuNPs, it was expected that the maximum absorbance should be at 520 nm with an extinction coefficient of 3.9 × 10^8^ M^−1^ cm^−1^ [56,57,58,59].

### 4.2. Protein Preparation

^15^N-labeled WT GB3 and its variant K19C GB3 were expressed and purified according to previously published methods [37,59,60]. Protein purity was established using SDS-PAGE electrophoresis. The H1.5 peptide (KVAKpSPKKAKAW, where pS represents phosphoserine) [61] and H1.5-Cys (KVAKpSPKKCKAW) were purchased from GenScript (Piscataway, NJ, USA) and BioMatik (Wilmington, DE, USA) at 95% purity and used after dissolving in appropriate buffer. GSH and BSA were purchased from Sigma Aldrich and were used without any additional purification. The concentration of GSH was determined using the molar mass; concentrations for all other peptides and proteins were determined using the calculated extinction coefficient at 280 nm [62].

### 4.3. PEGylated Gold Nanoparticle Preparation

The thiolated 5K, 10K, and 30K poly(ethylene glycol) monomethyl ether compounds (PEG-SH) were purchased from Laysan Bio, Inc. (Arab, AL, USA). The PEG-SH compounds were dissolved in MilliQ water containing 50 mM TCEP to maintain reducing conditions. A solution of 120 nM AuNPs was incubated with 100 μM of the three different sizes of PEG to fully saturate the AuNP surface. After the overnight incubation at room temperature, the solutions were centrifuged at 9000× *g* and washed three times with buffer to remove any unbound PEG molecules. Afterward, PEG-coated AuNPs were resuspended in buffer. The PEGylated AuNPs were characterized using UV-Vis spectroscopy and dynamic light scattering to confirm the functionalization. The same extinction coefficient was used for PEGylated AuNPs as for bare AuNPs. Atomic absorption spectroscopy confirmed that this approximation was accurate to within 6% error (Table S2). The PEG density on the AuNP surface was determined as follows. A total of 120 µM thiolated PEG was mixed with 120 nM 15-nm AuNPs. After 1-h incubation, all PEG bound AuNPs were thoroughly spun down (10 min at 21,000× *g*) and the bound fraction of PEG was determined by the reduction in thiol concentration in the supernatant using Ellman’s reagent (5,5′-dithio-bis (2-nitrobenzoic acid)) [63]. The coordination efficiency (θ) of PEG was calculated using θ = 1 − [SH]_free_/[SH]_total_, where [SH]_total_ and [SH]_free_ correspond to concentration of thiolated PEG in the supernatant before and after binding to AuNP. The PEG grafting density (ρ_PEG_) was calculated using formula ρ_PEG_ = θ ρ_graft_, where ρ_graft_ is the number of initial PEG molecules available per nm^2^ AuNP surface.

### 4.4. Transmission Electron Miscroscopy (TEM) Measurement of PEGylated AuNPs

Aliquots of 5 µL of 2 nM PEGylated AuNP solution was deposited on Formvar-coated copper grids. The excess liquid was wicked away, and the remaining thin film on the grid was allowed to dry. Prepared grids were imaged using JEOL 2100 with an accelerating voltage of 200 kV. TEM was performed at the Institute for Imaging and Analytical Technologies (I^2^AT) at Mississippi State University.

### 4.5. NMR Adsorption Measurements

For the control sample, 20 µM protein or peptide was prepared with 20 mM phosphate buffer at pH 6.5. Protein samples were mixed with AuNPs at concentration of 120 nM AuNPs. Quantitation was performed using an external standard, described previously [38]. This approach is effective when adsorption is slow on the NMR timescale, and when no line broadening is observed, as occurs here [38,50]. A solution of 50 mM TCEP and pH 6.5 PIPES buffer was used in the sample preparation of proteins and peptides containing thiol groups (GSH, H1.5-Cys, and K19C GB3). The samples were incubated for 6–72 hr before taking the 1D ^1^H NMR spectra for GSH, H1.5, and BSA. ^1^H-^15^N HSQC spectra were collected to measure the bound protein concentration of WT GB3 and K19C GB3. The NMR spectra were recorded at 25 °C using a 600 MHz Bruker Avance III cryoprobe-equipped NMR spectrometer. NMR spectra were processed using TOPSPIN 3.1 software. The bound protein concentration was measured by using the DSS peak as an external reference and integrating the protein amide signal with and without AuNPs at different time intervals [37,38]. First, all spectra were normalized to the external DSS reference peak. Then, the ratio of each peak intensity relative to the protein signal in the absence of nanoparticles was used to quantify the amount of protein remaining in solution. This ratio (r) represents the quantitative loss in protein signal, and in a standard 2D HSQC NMR spectrum can be averaged over all non-overlapping protein peaks. The concentration of free protein is then calculated as $\left(1-r\right)C_{0}$ where $C_{0}$ is the initial concentration of protein in the absence of AuNPs (here, $C_{0}$ is 20 µM). Additional details and experimental considerations are discussed in Xu et al. [38]. For 1D measurements (GSH, H1.5, H1.5-Cys, and BSA), the amide region of a water suppression experiment is used to calculate r for the entire amide proton region, as described in Wang et al. [37]. Examples of the 1D and HSQC NMR spectra are provided in the Supporting Information as Figures S3 and S4. Error bars are calculated as the standard error of the mean from at least three independent measurements.

### 4.6. Dynamic Light Scattering

A Wyatt DynaPro NanoStar DLS instrument was used to measure the NP size distributions. After equilibration for 1 h at room temperature, the solution was diluted 5-fold before transferring to a disposable microcuvette for measurement. The hydrodynamic diameters of the AuNPs were measured using the regularization fit functionality of the DYNAMICS software. For each measurement (with or without PEG), the average value of three independently prepared samples is reported and the uncertainty is calculated as the standard error of the mean. Zeta potential measurements were performed on an Anton Paar Litesizer 500 instrument using Kalliope software.

## Figures and Tables

**Table 1:** The hydrodynamic diameter (D_H_), localized surface plasmonic resonance (LSPR), Zeta potential and PEG density of AuNP and its three different conjugates of PEG.

AuNP-PEG Conjugate	D_H_ (nm)	Zeta Potential (mV)	LSPR Peak (nm)	PEG Density (PEG/nm^2^)
AuNP	18.4 ± 1.1	−38 ± 3	520	0
AuNP-PEG-5K	27.7 ± 0.5	−16.4 ± 0.8	523	0.96 ± 0.01
AuNP-PEG-10K	39 ± 2	−22.1 ± 0.6	522	0.67 ± 0.01
AuNP-PEG-30K	78 ± 3	−18 + 2	521	0.57 ± 0.01

**Table 2:** Characterization of size, number of surface cysteines and net charge of the ligands used in this work.

	GSH	H1.5	H1.5-Cys	WT GB3	K19C GB3	BSA
Size (kDa)	0.3	1.4	1.4	6.2	6.2	66
Surface cysteines	1	0	1	0	1	1
Estimated net charge at pH 7	−1	5	5	−2	−3	−17

## Data Availability

All data from this study are available in the figures and Supporting Information. Tabulated data points are available from the authors upon request.

## Supplementary Materials

The following are available online. Figure S1: UV-vis spectra of PEGylated AuNPs; Figure S2: Transmission electron microscopy (TEM) characterization of PEG-grafted gold nanoparticles; Figure S3: Example of quantifying protein unbound concentrations using 1D NMR for BSA; Figure S4: Example of quantifying protein bound concentrations using 2D NMR for K19C GB3; Table S1: Summary of bound concentration ([bound]) of ligand when mixing 20 μM ligand with 120 nM 15-nm AuNPs and observed rate constants (*k_obs_*) of 20 μM different ligands onto 120 nM PEGylated AuNPs used in this work; Table S2: Comparison of AuNP-PEG concentrations determined by atomic absorption spectroscopy (AAS) and concentrations determined using the extinction coefficient at 520 nm (3.9 × 10^8^ M^−1^ cm^−1^), as described in the text.

## Sample Availability

Protein expression vectors for GB3 are available from the authors upon request. All other proteins and peptides are available commercially.
