# Supplementary material for: Understanding the Adsorption of Peptides and Proteins onto PEGylated Gold Nanoparticles
Source: Molecules. 2021 Sep 24;26(19):5788. doi: 10.3390/molecules26195788 (PMC8510204; doi:10.3390/molecules26195788)
Supplement: Supplementary file 1 [file molecules-26-05788-s001.zip › molecules-1340392-supplementary.pdf]

Supporting Information for:

Understanding the Adsorption of Peptides and Proteins onto PEGylated Gold Nanoparticles

Y. Randika Perera,[1,†] Joanna Xiuzhu Xu, [1,†] Dhanush L. Amarasekara,[1] Alex C. Hughes,[1] Ibraheem Abbood,[2] and Nicholas C. Fitzkee[1,*]

[1] Department of Chemistry, Mississippi State University, Mississippi State, Mississippi 39762, United States

[2] Department of Chemistry, University of Arkansas at Little Rock, Little Rock, Arkansas 72204, United States

[†] These authors contributed equally to this work.

* To whom correspondence should be addressed:

Email: nfitzkee@chemistry.msstate.edu

ORCiD: 0000-0002-8993-2140

Phone: (662) 325-1288

Fax: (662) 325-1618

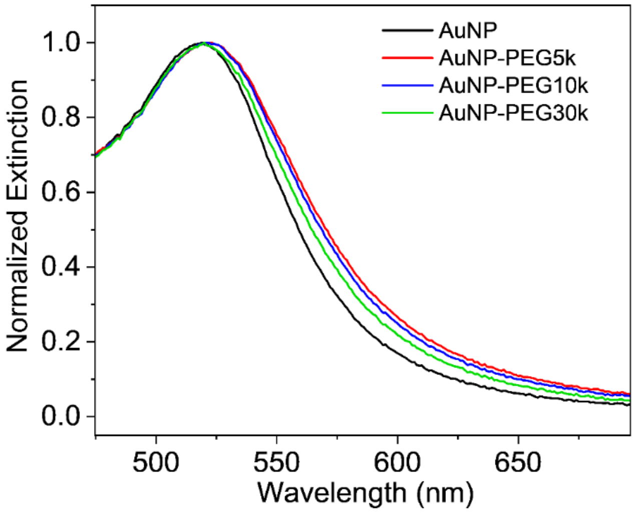

Figure S1. UV-vis spectra of (black) bare AuNP, (red) 5K-PEG-SH coated AuNP, (blue) 10K-PEG-SH coated AuNP, (green) 30K-PEG-SH coated AuNP. All spectra are normalized with the maximum extinction to be 1.

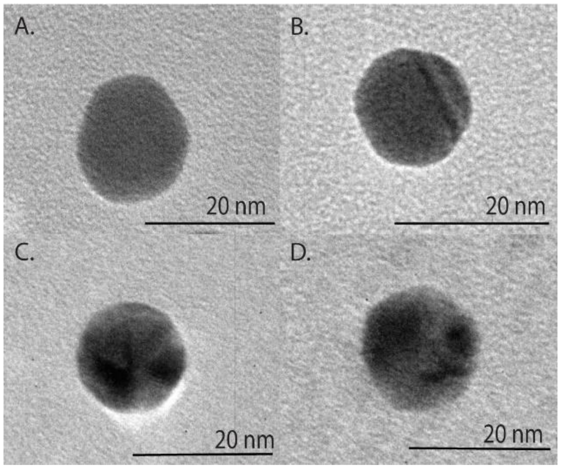

Figure S2. Transmission electron microscopy (TEM) characterization of PEG-grafted gold nanoparticles. Representative TEM images of (A) AuNP-PEG-5K, (B) AuNP-PEG-10K, (C) AuNP-PEG-30K and (D) bare 15 nm-AuNP.

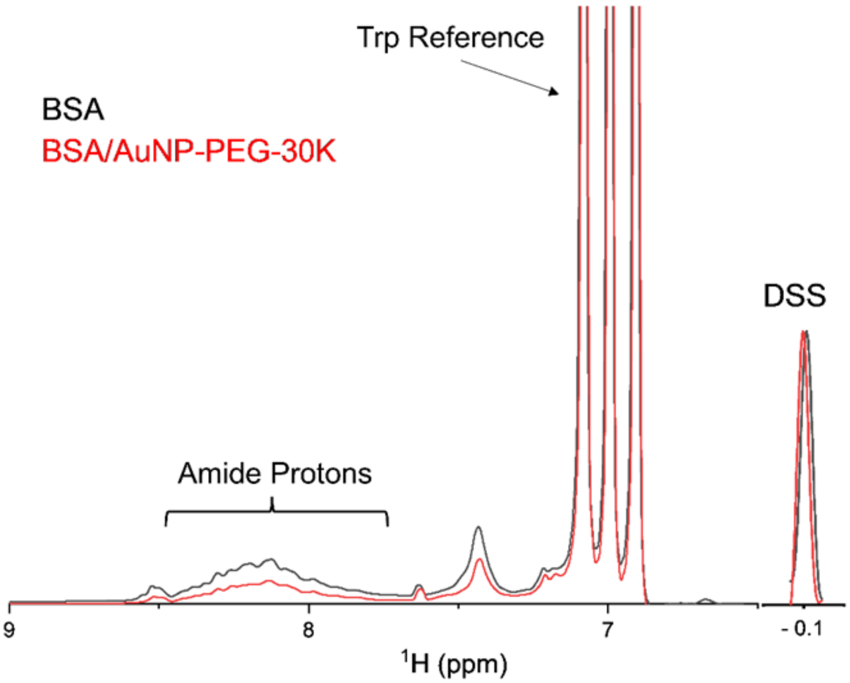

Figure S3. Example of quantifying protein unbound concentrations using 1D NMR for BSA. The black and red spectra are 20 μM BSA control sample and 20 μM BSA mixed with 120 nM AuNP-PEG-30K, respectively. Protein signals are reduced due to NP binding. TopSpin software is used to scale the amide proton region to calculate the amount of signal lost as a function of time.

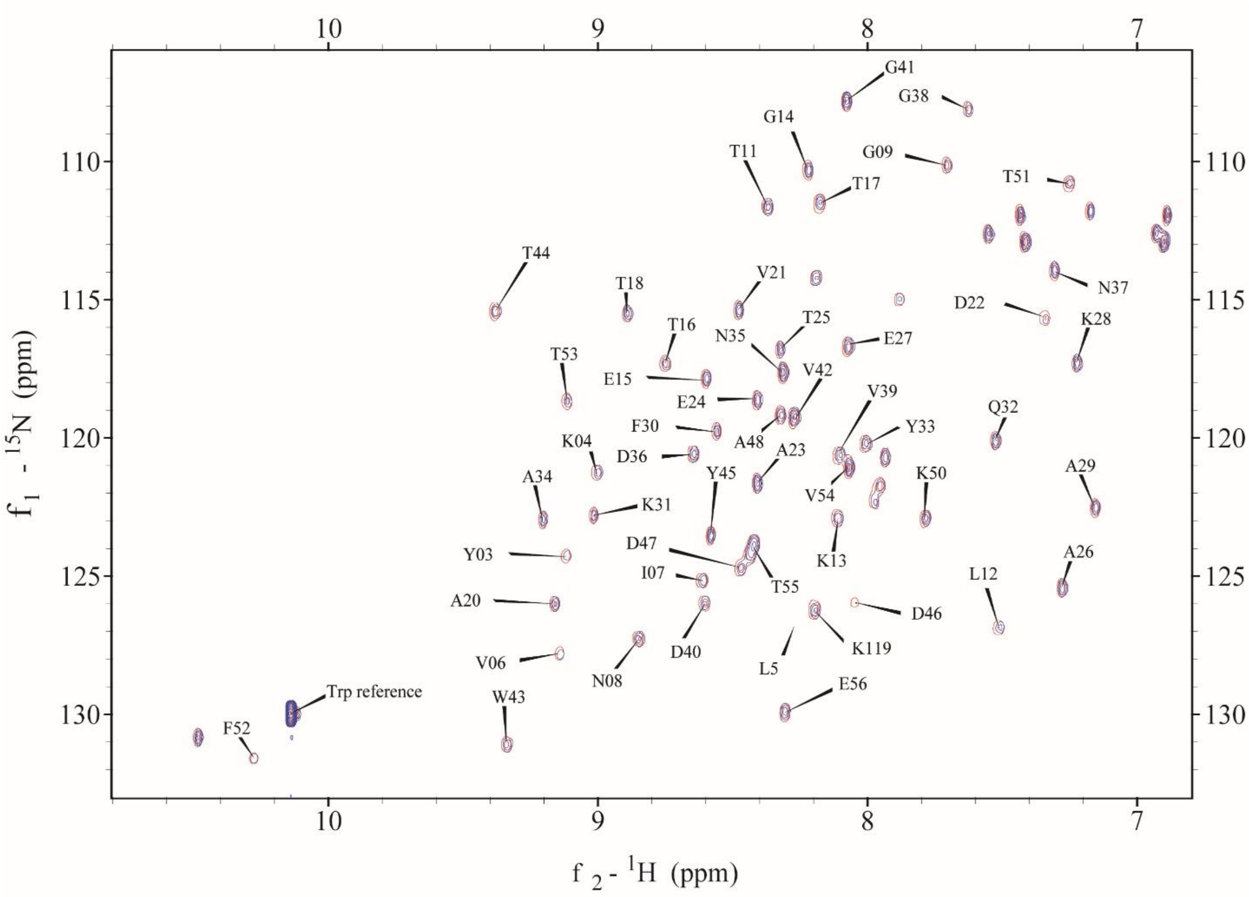

Figure S4. Example of quantifying protein bound concentrations using 2D NMR for K19C GB3. The red and blue spectra are 20 μM ^{15}N labeled K19C GB3 control sample and 20 μM K19C GB3 mixed with 120 nM AuNP-PEG-30K for 1 hr, respectively. Protein signals are reduced due to NP binding. The peak intensities of K19C GB3 control sample and NP-containing sample are first normalized by the external reference ^{15}N-Trp before determination of the fraction of unbound protein by dividing the latter by the former sample intensities.

Table S1. Summary of bound concentration ([bound]) of ligand when mixing 20 μM ligand with 120 nM 15-nm AuNPs and observed rate constants (k_{obs}) of 20 μM different ligands onto 120 nM PEGylated AuNPs used in this work

	**AuNP-PEG-5K**		**AuNP-PEG-10K**		**AuNP-PEG-30K**	
	**[bound] (μM)**	$k_{obs} \times 10^{-5}$ **(s⁻¹)**	**[bound] (μM)**	$k_{obs} \times 10^{-5}$ **(s⁻¹)**	**[bound] (μM)**	$k_{obs} \times 10^{-5}$ **(s⁻¹)**
**H1.5**	6.8 ± 0.5	1.3 ± 0.2	9.7 ± 0.2	3.0 ± 0.5	12.8 ± 0.3	4.3 ± 0.2
**H1.5-Cys***	17.7 ± 0.2	460 ± 80	18.4 ± 0.2	690 ± 120	18.0 ± 0.2	1390 ± 70
**GSH**	9.6 ± 0.4	2.9 ± 0.1	11.7 ± 0.1	4.0 ± 0.2	14.8 ± 0.2	6.1 ± 0.2
**wt GB3**	0.4 ± 0.5	N/A	2.4 ± 0.4	1.0 ± 0.5	4.9 ± 0.5	1.9 ± 0.8
**K19C GB3**	2.0 ± 0.5	5.4 ± 0.5	4.7 ± 0.4	9.0 ± 1.4	7.0 ± 0.4	44 ± 7
**BSA**	2.0 ± 0.4	7.6 ± 1.8	2.2 ± 0.4	15.0 ± 1.9	3.2 ± 0.2	14 ± 2

* The fastest rate observable in our experiments is approximately 900 s^{-1} based on a 15-minute dead time. The rates for H1.5-Cys are therefore approximate.

Table S2. Comparison of AuNP-PEG concentrations determined by atomic absorption spectroscopy (AAS) and concentrations determined using the extinction coefficient at 520 nm (3.9×10^8 M^{-1} cm^{-1}), as described in the text.

	**[AuNP] by AAS (nM)**	**[AuNP] by UV-vis (nM)**	**Difference (%)**
**AuNP**	1.638	1.60	2.5
**AuNP-PEG-5K**	1.638	1.69	-3.2
**AuNP-PEG-10K**	1.637	1.73	-5.8
**AuNP-PEG-30K**	1.637	1.67	-1.8
